# Supplementary material for: Virus Adaptation Following Experimental Infection of Chickens with a Domestic Duck Low Pathogenic Avian Influenza Isolate from the 2017 USA H7N9 Outbreak Identifies Polymorphic Mutations in Multiple Gene Segments
Source: Viruses. 2021 Jun 18;13(6):1166. doi: 10.3390/v13061166 (PMC8234733; doi:10.3390/v13061166)
Supplement: Supplementary file 1 [file viruses-13-01166-s001.zip › viruses-1261018-supplementary.pdf]

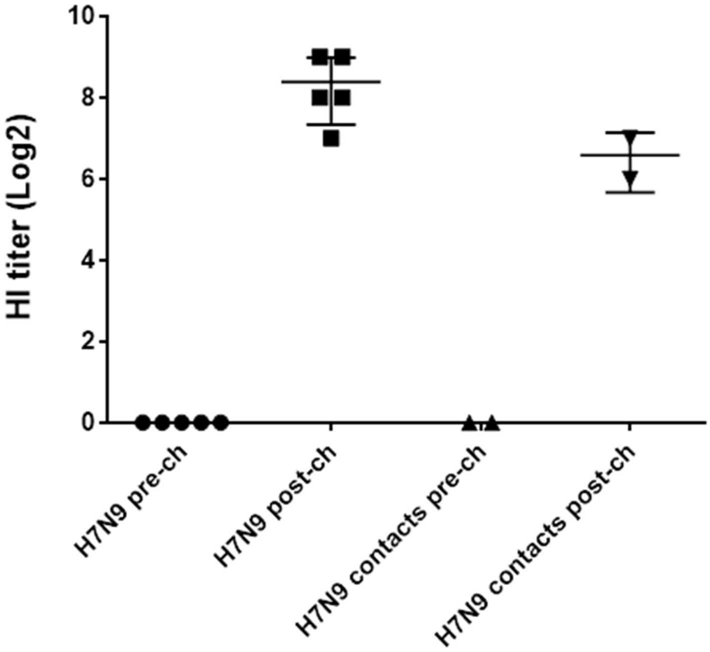

Figure S1. Individual HI titers (log2) and standard error for bird groups against A/duck/AL/2017 (H7N9) challenge virus; pre challenge (pre-ch) and two weeks post-challenge (post-ch).

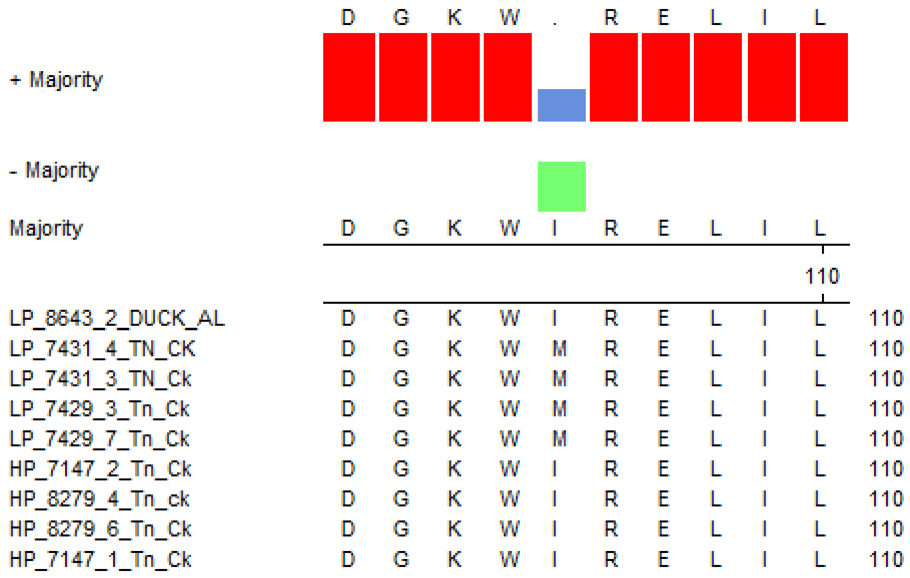

Figure S2. Alignment of nucleoprotein segments of low pathogenic avian influenza (LPAI) and highly pathogenic avian influenza (HPAI) viruses of H7N9 subtypes recovered from clinical specimens of chicken and duck obtained during the 2017 U.S. outbreak. The changes at position 105 in NP proteins are demonstrated. GenBank accession numbers: MF357837.1, KY818820.1, MF357789.1, MF357797.1, KY818812.1, KY818836.1, MF357829.1, MF357741.1.

Table S1: The synonymous changes found in A/duck/AL/2017 (H7N9) viral genome segments recovered from cloacal swab samples. The frequencies of variants were computed using with minimum sequence coverage of 500 reads cover the base, and minimum variant frequency of 5%. The maximum P-value of 10-6 and minimum strand-bias p-value of 10-5 when exceeding 65% bias were used to call the variants with average quality >35.

Segment	CDS Codon Number	Change	Codon Change	Variant Frequency	Coverage
PB2	67	C -> A	AUC -> AUA	12.5	7676
	74	G -> A	GGG -> GGA	5	5704
	412	G -> A	AAG -> AAA	16.3	1046
	637	T -> A	ACU -> ACA	6.6	1100
	643	A -> C	UCA -> UCC	7.7	2302
	662	C -> A	ACC -> ACA	8.5	9170
	666	A -> T	ACA -> ACT	7.1	5883
PB1	255	T -> A	GUU -> GUA	65.5	1283
	257	A -> G	ACA -> ACG	8.8	1047
	472	G -> A	CUG -> CUA	19.8	4084
	582	A -> G	CAA -> CAG	6.4	1802
PA	92	C -> T	AAC -> AAT	6.3	2576
	100	C -> T	GUC -> GUT	5.5	6202
	553	A -> G	GCA -> GCG	6.5	1587
NP	410	C -> T	CCC -> CCT	8.5	21779
NS	79	G -> T	CUG -> CUT	8.4	17513
	131	A -> G	AAA -> AAG	18.8	19188
